# Pre-vaccination biomarkers of enteric dysfunction, inflammation and nutritional status associate with seroconversion to oral cholera vaccine and are perturbed during natural infection

**DOI:** 10.3389/fimmu.2026.1917668

**Published:** 2026-08-14

**Authors:** Fraser Liswaniso, Bernard Phiri, Mwelwa Chibuye, Charlie C. Luchen, Dennis Ngosa, Harriet Ng’ombe, Kennedy Chibesa, Mutinta Muchimba, Roma Chilengi, Samuel Bosomprah, Caroline C. Chisenga

**Affiliations:** 1 Basic Sciences and Immunology, Centre for Infectious Disease Research in Zambia (CIDRZ), Lusaka, Zambia; 2 Department of Epidemiology and Biostatistics, University of Zambia, Lusaka, Zambia; 3 Amsterdam University Medical Centers (UMC), location University of Amsterdam, Department of Global Health, Amsterdam Institute for Global Health and Development, Amsterdam, Netherlands; 4 Amsterdam Institute of Infection and Immunity, Amsterdam University Medical Centers, Amsterdam, Netherlands; 5 Centre for Epidemic Response and Innovation, University of Stellenbosch, Stellenbosch, South Africa; 6 Zambia National Public Health Institute (ZNPHI), Lusaka, Zambia; 7 Department of Biostatistics, School of Public Health, University of Ghana, Accra, Ghana; 8 Parasites and Microbes Programme, Wellcome Sanger Institute, Hinxton, United Kingdom

**Keywords:** biomarkers, cholera, MEEDAT, thyroglobulin, vaccine

## Abstract

**Background:**

Oral cholera vaccines (OCVs) show variable immunogenicity in endemic settings. Host factors including enteric dysfunction, systemic inflammation, and nutritional status may influence responses, yet their role in shaping OCV immune responses is not fully understood. This study examined associations between pre-vaccination biomarkers spanning these domains and vibriocidal seroconversion to an OCV, while characterizing how the same biomarker profiles are altered during acute natural *V. cholerae* infection.

**Methods:**

We conducted a retrospective analysis of stored samples from two Zambian cohorts. The primary cohort comprised 225 adults vaccinated with Shanchol™ IN 2021.The comparator cohort included 38 individuals with acute *V. cholerae* infection during the 2023 outbreak. Eleven biomarkers were measured using the MEEDAT 11-plex ELISA, and vibriocidal antibodies were assessed. Associations with seroconversion were assessed using cross-fit partialling-out LASSO Poisson regression and quartile analysis. Biomarker profiles were compared between cohorts using Wilcoxon rank-sum and Spearman correlation tests.

**Results:**

Quartile analysis showed higher seroconversion rates among participants with low to mid-range thyroglobulin levels, while the highest quartile had reduced responses (Inaba Q4: 36.2%; Ogawa Q4: 49.2%). In multivariable LASSO Poisson regression, higher baseline FGF-21 was independently associated with increased Inaba seroconversion (adjusted IRR 1.10, 95% CI 1.04-1.17, p=0.001). Compared with the pre-vaccination baseline, acutely infected individuals exhibited lower ferritin, IGF-1 and RBP4, and higher sTfR, CRP, AGP and FGF-21, together with a restructured inter-biomarker correlation network.

**Conclusion:**

Selected pre-vaccination host biomarkers, particularly FGF-21 and thyroglobulin, are associated with OCV seroconversion. The profound multi-domain perturbation observed during acute infection underscores the importance of measuring these markers under stable baseline conditions when studying vaccine immunogenicity.

## 1 Introduction

Cholera continues to pose a significant public health challenge in many low- and middle-income countries, disproportionately impacting vulnerable communities in endemic and outbreak-prone locations. According to the most recent Global Burden of Disease (GBD) estimates, cholera accounted for approximately 86,500 deaths (95% UI: 69,700–105,000) in 2021 (1). Oral cholera vaccination is now widely regarded as a key intervention for effectively controlling and preventing the disease (2). These vaccines primarily confer protection by inducing a localised immune response in the gut, which reduces the risk of symptomatic cholera and may, in some contexts, contribute to reduced transmission by limiting bacterial shedding. Although Shanchol™ and other oral cholera vaccines can confer population-level protection and demonstrate good immunogenicity in some settings (3, 4), other studies report poor immune responses, rapid antibody waning, and limited boosting following revaccination (5, 6).

Host physiological factors common in low-resource settings have been proposed as potential contributors to suboptimal responses to oral vaccines (7). These include systemic inflammation, nutritional deficiencies, and chronic enteric insults such as Environmental Enteric Dysfunction (EED), a subclinical condition characterized by altered structure and function of the small intestine (8–10). However, most existing studies have examined these factors in isolation, often using limited biomarker panels or non-functional immunologic outcomes. Markers of intestinal epithelial damage and microbial translocation (such as I-FABP and sCD14) capture the structural and functional consequences of environmental enteric dysfunction (1), while acute-phase proteins (CRP and AGP) reflect the chronic inflammatory milieu that can suppress vaccine responses (8). Growth and metabolic stress markers, including IGF-1 and FGF-21, provide insight into the endocrine–immune interactions that influence lymphocyte function (2), and micronutrient indicators (ferritin, sTfR, RBP4, and thyroglobulin) reflect deficiencies known to compromise both innate and adaptive immunity (11). Measuring these domains together allows a more integrated assessment of the host physiological constraints that may limit OCV performance in endemic adult populations.

Few studies have taken an integrated approach to evaluate how multiple host biomarker domains collectively relate to functional immune responses to oral cholera vaccination, particularly in cholera-endemic adult populations.

A critical but underappreciated challenge in this area is that the biomarkers used to characterize host physiological status are not static, they are highly sensitive to acute infectious stress (12). In cholera-endemic settings, where repeated enteric exposures are common, the same biomarkers that reflect a patient’s baseline nutritional or inflammatory state may also be transiently and substantially perturbed by active infection (13). Understanding how acute cholera infection reshapes the host biomarker environment is therefore not a separate question. It is essential context for interpreting what biomarker profiles measured at vaccination baseline represent, and why certain biomarker–vaccine associations may emerge or be obscured in endemic populations.

This study aimed to characterize the host biomarker conditions that support or hinder immune responses to oral cholera vaccination in a cholera-endemic adult population in Zambia by examining associations between pre-vaccination baseline biomarkers spanning enteric dysfunction, systemic inflammation, growth factors, and micronutrient status and vibriocidal seroconversion following OCV.

## 2 Materials and methods

### 2.1 Study design and participants

This was a retrospective study comprising analysis of secondary data from two complementary sources. The primary analytic cohort comprised 225 adults sampled from Lukanga Swamps, In the central province of Zambia who were vaccinated with Shanchol™ oral cholera vaccine (Shantha Biotechnics Private Limited, Hyderabad, India) during the 2021 cholera outbreak and followed up at five time points over 90 days. All 225 individuals reported a history of receiving Shanchol during the 2016 cholera outbreak; the number of doses received in 2016 (0, 1 or 2) is recorded as ‘Vaccination History’ in **Table 1**.

**Table 1:** Baseline characteristics of revaccinated individuals.

Characteristic	Total n=212
	n (% of total)
Sex	
Male	88 (41.5)
Female	124 (58.5)
Age category	
15–25	64 (30.2)
26–45	71 (33.5)
46+	69 (32.5)
Missing	8 (3.8)
HIV status	
Negative	170 (80.2)
Positive	41 (19.3)
Missing	1 (0.5)
**Baseline Inaba titre, Median (IQR)**	7.5 (5–40)
**Baseline Ogawa titre, Median (IQR)**	10 (5–80)
Vaccination history	
0 Doses	62 (29.2)
1 Dose	86 (40.6)
2 Doses	64 (30.2)

Bold values indicate the largest category within each variable (or median baseline titres where applicable).

Inclusion criteria: adults aged 15–65 years residing in a cholera hotspot area who received Shanchol in 2021 and had previously received Shanchol in 2016. Exclusion criteria: incomplete paired baseline and post-vaccination vibriocidal titre data, or insufficient sample volume for biomarker analysis.

Blood samples were collected at baseline (day 1) prior to the first dose, at day 14 (prior to the second dose), and at days 28, 60, and 90 post-dose 1. Biomarker profiles from pre-vaccination baseline samples were used in the primary analysis of associations with vibriocidal seroconversion (n=212 with complete seroconversion data).

In the secondary analysis, the same baseline biomarker profiles were compared to biomarker profiles measured during acute natural cholera infection from the comparator cohort. This comparator cohort comprised 38 adults with laboratory-confirmed acute *V. cholerae* infection recruited during the 2023–2024 cholera outbreak in Lusaka. Inclusion criteria were adults aged 15–65 years admitted to designated cholera treatment centres with laboratory-confirmed *V. cholerae* infection. Exclusion criteria were inability to provide informed consent or insufficient sample volume for biomarker analysis.

This comparison characterizes the extent to which acute cholera infection perturbs the host biomarker environment relative to the endemic-population baseline, providing critical interpretive context for the biomarker–vaccine associations observed in the primary analysis.

### 2.2 Sample collection and processing - revaccinated cohort (cohort 1)

Approximately 10 mL of blood was collected from adults aged 15–65 residing in a cholera hotspot area in the Central Province of Zambia. Baseline samples were obtained prior to administration of the first dose of Shanchol™. Blood was centrifuged at 3,500 RPM for 5 minutes to separate plasma and serum. All samples were stored at −80 °C until analysis. Primary cohort samples were stored for 10–16 months prior to laboratory testing.

### 2.3 Sample collection and processing - naturally infected cohort (cohort 2)

Blood samples were collected during the acute infection phase from clinically confirmed cholera patients admitted to designated cholera treatment centres in Lusaka (George Health Centre, Matero Level I Hospital, Heroes Stadium, and Levy Mwanawasa Hospital) during the 2023/2024 outbreak. After obtaining informed consent, approximately 10 mL of blood was drawn into EDTA and SST tubes. Plasma and serum were separated, aliquoted, and transported to the CIDRZ laboratory at 2–8 °C. All samples were stored at −80 °C until analysis. Acute-infection cohort samples were stored for 6–15 months prior to laboratory testing.

### 2.4 Laboratory assays

#### 2.4.1 Measurement and quantification of biomarkers

A total of 11 biomarkers were quantitatively analysed using the MEEDAT 11-plex ELISA (Q-Plex™ Human Environmental Enteric Dysfunction 11-plex, Quansys Biosciences, USA). These included proxies for EED (Intestinal Fatty Acid-Binding Protein [I-FABP] and Soluble CD14 [sCD14]), growth resistance (Insulin-Like Growth Factor 1 [IGF-1] and Fibroblast Growth Factor 21 [FGF-21]), systemic inflammation (C-Reactive Protein [CRP] and α1-acid glycoprotein [AGP]), micronutrient status (Ferritin, Soluble Transferrin Receptor [sTfR], Retinol Binding Protein 4 [RBP4], and Thyroglobulin [Tg]), and malaria antigenemia (Histidine-Rich Protein 2 [HRP2]). All analytes were measured in accordance with methods described by Arndt et al. (14).

Briefly, an 8-point calibration curve was prepared using serial dilutions of the reconstituted calibrator in sample diluent. Plasma samples were diluted 1:10 in sample diluent, with further dilution for high analyte concentrations. Fifty μL of standards and samples were loaded in duplicate into a Q-Plex™ Array 96-well plate and incubated at 500 RPM for 2 hours at room temperature. After washing, 50 μL of Detection Mix was added, followed by incubation for 1 hour, further washing, addition of 50 μL Streptavidin-HRP, and 20-minute incubation. After final washes, 50 μL substrate was added and the plate was imaged within 5 minutes. Intra- and inter-assay coefficients of variation (CV) were maintained at <10% and <15%, respectively. Results were processed and exported using Quansys Q-View™ software (version 3.11).

#### 2.4.2 Measurement of vibriocidal antibody responses

Vibriocidal antibody titres were measured in all serum samples using a functional assay with Guinea pig complement (Merck, Darmstadt, Germany) and the target strains V. cholerae O1 Ogawa PIC158 and Inaba PIC018 (15). Colonies from overnight cultures were inoculated into Brain Heart Infusion (BHI) broth and incubated for approximately 4 hours at 37 °C. Heat-inactivated serum, exogenous guinea pig complement (Sigma Aldrich S1639-5ML), and V. cholerae bacterial cells were added to 96-well microtiter plates and incubated at 37 °C. The reciprocal of the maximum serum dilution producing a 50% decrease in optical density at 595 nm compared to positive control wells was used to calculate vibriocidal titres. A fourfold or greater rise in vibriocidal titres from baseline was considered seroconversion (16).

### 2.5 Variables

The primary outcome was seroconversion, defined as a fourfold increase in vibriocidal titres between pre-vaccination baseline and day 14 and day 28 post-vaccination (6). Previous vaccination dose, age, sex, and HIV status were considered confounders based on subject-matter knowledge and were adjusted for in all regression models.

### 2.6 Statistical analysis

Baseline characteristics were summarised using frequency and percentage for categorical variables and median with interquartile range (IQR) for continuous variables. Associations between baseline biomarkers and seroconversion were evaluated using a partialling-out LASSO Poisson regression model with robust standard errors, adjusting for age, sex, HIV status, and previous vaccination status. Participants with missing seroconversion data (n = 13) were excluded from the primary analyses, no imputation was performed. Biomarker titres were normalised (mean=0, SD = 1) to permit interpretation of effect sizes as one standard deviation increase in baseline biomarker concentration. In secondary analyses, biomarkers were modelled as quartile-defined categorical variables.

To contextualise the biomarker–vaccine findings, biomarker concentrations were compared between the pre-vaccination baseline cohort (n=225) and the acutely infected comparator cohort (n=38) using the Wilcoxon rank-sum test, with results presented as median (IQR). Pairwise Spearman correlation analyses were conducted within each group to examine inter-biomarker relationships and assess how acute infection restructures the host biomarker network. All analyses were conducted in Stata 18 (StataCorp, College Station, TX, USA). Graphs were created in R software. Statistical significance was set at α=0.05.

## 3 Results

### 3.1 Participant flow and baseline characteristics of vaccinated individuals

**Figure 1:**
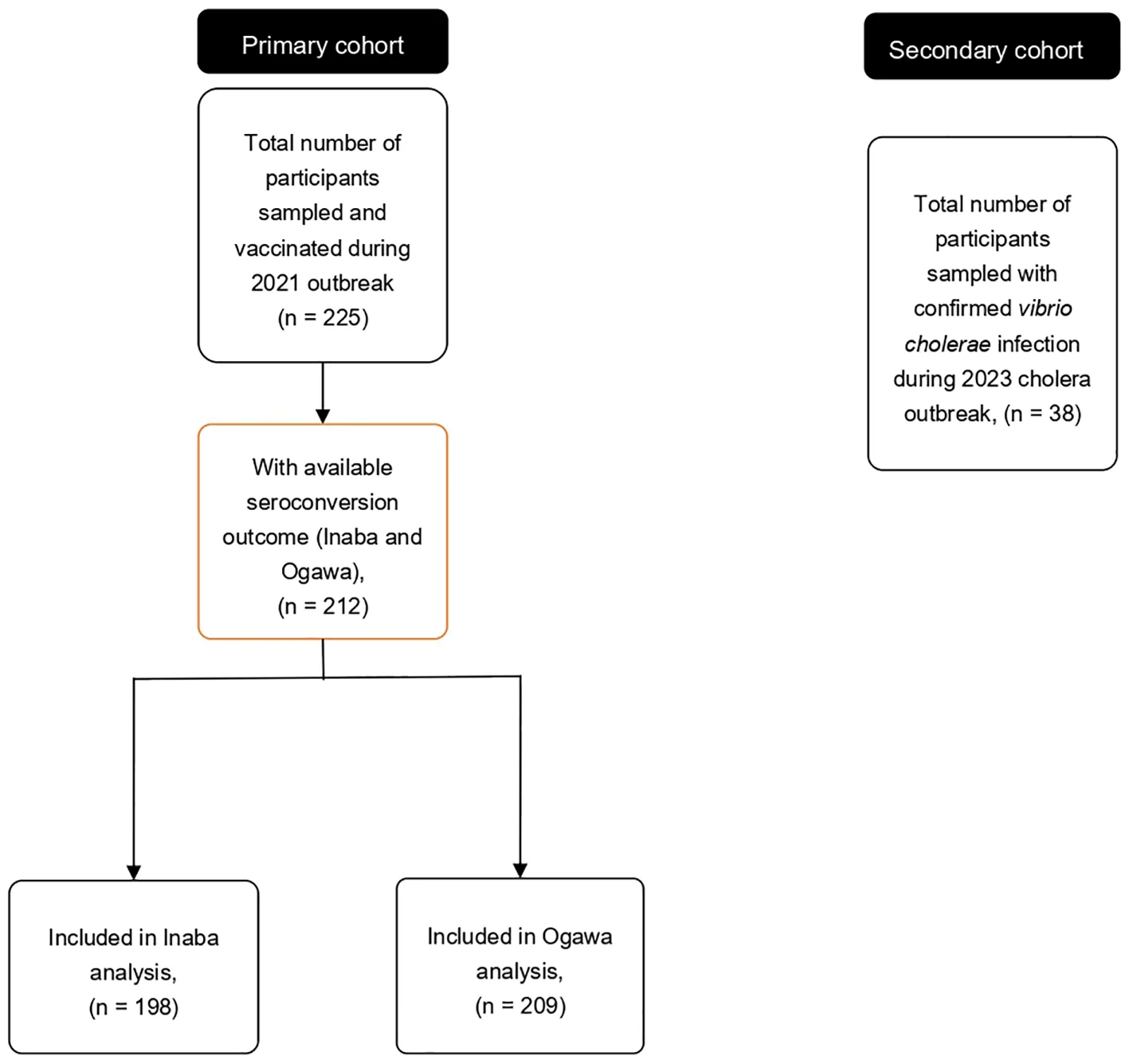
Study flow chart.

A total of 225 individuals were vaccinated, of whom 212 had seroconversion data and were included in the final analysis (**Figure 1**). Of the 212, 209 were included in Ogawa analyses and 198 in Inaba analyses due to missing seroconversion data for three individuals each.

The revaccinated individuals were predominantly female (58.5%), with the largest proportion aged 26–45 years (33.5%). Most participants were HIV-negative (80.2%). Baseline vibriocidal antibody titres were generally low to moderate, with median titres of 7.5 (IQR 5-40) for Inaba and 10 (IQR 5-80) for Ogawa. Vaccination history varied: 29.2% reported zero prior doses, 40.6% one dose, and 30.2% two doses (**Table 1**).

### 3.2 Association of biomarkers with seroconversion to Inaba and Ogawa *V. cholerae* strains

Of the 198 individuals with Inaba seroconversion data, 50.5% seroconverted; of the 209 with Ogawa data, 52.6% seroconverted. In adjusted LASSO Poisson regression analyses, most biomarkers showed no significant associations with seroconversion after accounting for previous vaccination dose, age, sex, and HIV status.

A notable exception was FGF-21 in the Inaba stratum. Higher FGF-21 levels were independently associated with increased Inaba seroconversion (IRR; 1.10, 95% CI: 1.04-1.17; p=0.001). No comparable significant association was observed for Ogawa seroconversion (**Table 2**).

**Table 2:** Associations of biomarkers with seroconversion among revaccinated individuals (LASSO Poisson regression).

Biomarker	Inaba. IRR (95% CI)	P-value	Ogawa. IRR (95% CI)	P-value
Nutritional status				
Ferritin	1.02 (0.86, 1.20)	0.855	0.98 (0.83, 1.16)	0.825
Tg	0.92 (0.75, 1.12)	0.401	0.99 (0.87, 1.14)	0.952
sTfR	0.92 (0.79, 1.08)	0.318	1.02 (0.92, 1.15)	0.660
RBP4	0.96 (0.80, 1.16)	0.663	1.01 (0.85, 1.21)	0.828
Malaria				
HRP2	1.04 (0.93, 1.15)	0.512	1.02 (0.91, 1.15)	0.704
Growth factors				
IGF-1	0.95 (0.80, 1.13)	0.555	0.98 (0.85, 1.14)	0.823
FGF-21	1.10 (1.04, 1.17)	0.001*	1.05 (0.96, 1.16)	0.272
I-FABP	0.94 (0.79, 1.12)	0.490	0.86 (0.73, 1.02)	0.081
sCD14	1.00 (0.83, 1.20)	0.996	0.97 (0.82, 1.16)	0.772
Systemic inflammation				
CRP	0.95 (0.79, 1.14)	0.593	1.07 (0.94, 1.23)	0.299
AGP	1.12 (0.91, 1.39)	0.295	0.99 (0.82, 1.20)	0.958

*p<0.05. Adjusted for previous vaccination dose, age category, sex, and HIV status. Ogawa n=209, Inaba n=198.

In quartile analyses, thyroglobulin levels showed a non-linear association with seroconversion. Mid-range concentrations (Q2 and Q3) were associated with higher seroconversion likelihood, while the highest quartile (Q4) was associated with the lowest rates for both serotypes (Inaba p=0.006; Ogawa p=0.003) (**Supplementary Table 1**). No consistent or significant trends were observed for growth factor, EED, or inflammatory biomarkers across quartiles (**Supplementary Tables S2**, **S3**).

### 3.3 Alterations in biomarker levels and inter-relationships during acute cholera infection

To contextualise the biomarker profiles observed in the primary cohort, we compared pre-vaccination baseline biomarker concentrations from the full vaccinated cohort (n=225) against biomarker profiles measured during acute infection (n=38). This comparison characterizes the extent to which acute cholera infection perturbs the same biomarker domains relevant to vaccine immune responses. The characteristics of the two groups are shown in **Supplementary Table 4**.

**Figure 2:**
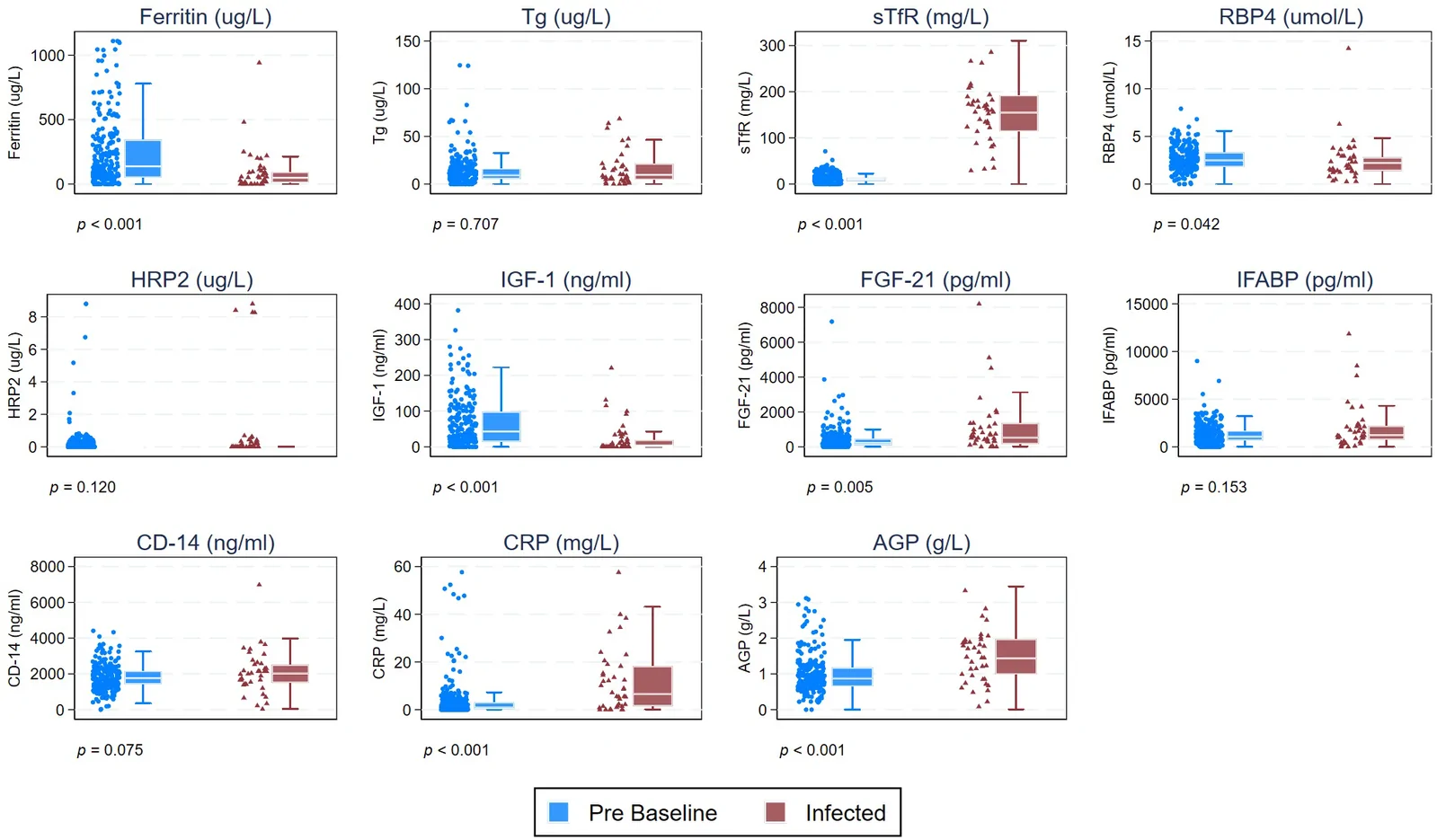
Comparison of biomarker concentrations between the pre-vaccination baseline cohort (n=225) and naturally infected individuals (n=38). Boxes represent the interquartile range; horizontal lines indicate the median. Significance indicated by Wilcoxon rank-sum test p-values.

Compared with the pre-vaccination baseline group (n=225; all individuals regardless of seroconversion data availability), acutely infected individuals (n=38) had significantly lower ferritin (p<0.001) and IGF-1 (p<0.001), and significantly higher sTfR (p<0.001), CRP (p<0.001), AGP (p<0.001), and FGF-21 (p=0.005). No significant differences were observed for RBP4, thyroglobulin, HRP2, I-FABP, or sCD14 (**Figure 2**).

**Figure 3:**
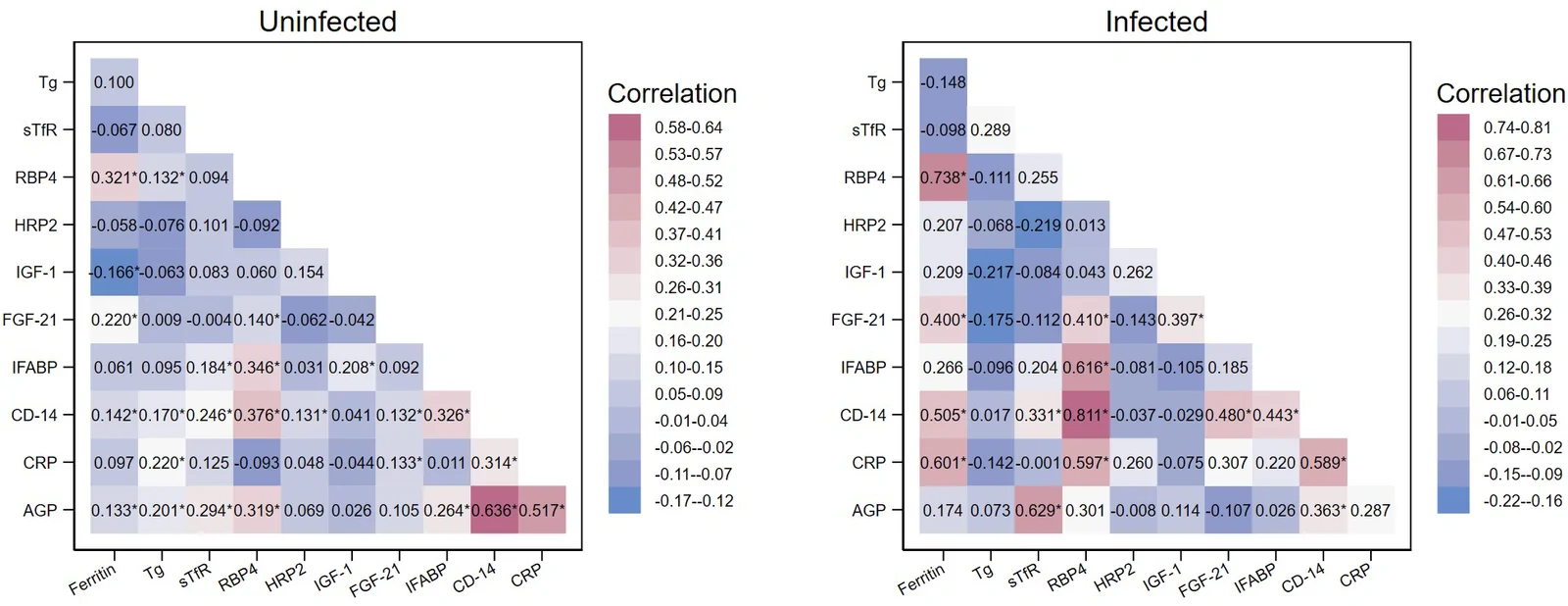
Spearman correlation matrices of biomarker inter-relationships in the pre-vaccination baseline group (left) and the acutely infected group (right). Colour intensity represents correlation strength; asterisks indicate statistical significance.

Pairwise Spearman correlation analysis revealed that acute infection substantially restructured inter-biomarker relationships relative to the pre-vaccination baseline (**Figure 3**). In the infected group, several strong and statistically significant correlations were present that were absent or substantially weaker in the pre-baseline group. These included RBP4 with ferritin (r=0.738), RBP4 with sCD14 (r=0.811), RBP4 with I-FABP (r=0.616), CRP with ferritin (r=0.601), CRP with sCD14 (r=0.589), AGP with sTfR (r=0.629), and FGF-21 with RBP4 (r=0.410). In contrast, the pre-vaccination baseline group showed sparse and modest inter-biomarker associations, limited to AGP with CRP (r=0.517) and AGP with sCD14 (r=0.636), consistent with a relatively undisturbed physiological state.

## 4 Discussion

Oral cholera vaccines have demonstrated variable efficacy in endemic settings, and host nutritional, metabolic, and inflammatory status are increasingly recognized as important modifiers of vaccine-induced immunity. This study pursued two linked objectives: first, to identify baseline host biomarker profiles that associate with seroconversion following OCV revaccination in a cholera-endemic Zambian cohort; and second, to characterize how the same biomarkers are perturbed during acute *V. cholerae* infection, providing a pathophysiologically relevant comparator.

Quartile analysis revealed a non-linear, threshold pattern: seroconversion rates were higher among participants with lower to mid-range thyroglobulin concentrations, while those in the highest quartile (Q4) had the lowest seroconversion for both serotypes (Inaba Q4: 36.2%; Ogawa Q4: 49.2%). This finding suggests that thyroid-metabolic stress beyond a certain threshold constrains OCV immune responsiveness. Although thyroglobulin did not reach statistical significance in the primary LASSO Poisson regression, the consistent quartile gradient across both serotypes warrants biological interpretation. Thyroglobulin is sensitive to iodine status, thyroid hormone synthesis, and inflammatory stress (17). In Zambia, mild to moderate iodine insufficiency persists in parts of the population despite salt iodisation efforts (18), so participants in the highest Tg quartiles may represent those with subclinical iodine insufficiency, non-thyroidal illness, or thyroid autoimmunity, any of which could impair immune priming and reduce OCV seroconversion. Thyroid hormones directly regulate lymphocyte proliferation, cytokine production, and macrophage activity (11), providing a plausible mechanistic pathway for thyroid-immune interaction. However, without TSH, free T4, or anti-Tg antibody measurements, the precise aetiology of elevated Tg in this cohort cannot be determined. This association should therefore be interpreted as a hypothesis-generating signal warranting prospective investigation.

In the primary multivariable LASSO Poisson regression, higher pre-vaccination FGF-21 was significantly associated with increased Inaba seroconversion, but not Ogawa seroconversion. FGF-21 is a hepatokine involved in energy homeostasis, insulin sensitivity, and lipid metabolism (19) and has been implicated in the activation of innate immune pathways through modulation of cytokine responses (20). Recent experimental evidence further indicates that FGF-21 can protect against macrophage-mediated inflammation and modulate innate immune responses under conditions of metabolic stress (21, 22).

The serotype-specific nature of this association is intriguing; Inaba and Ogawa vibriocidal responses may engage partially distinct mucosal and systemic immune effectors, and FGF-21-related metabolic signalling pathways may preferentially amplify one arm of this immune response. Our infection comparator data show that FGF-21 was significantly elevated during acute cholera, consistent with its role as an inflammation-responsive hepatokine released during sepsis and critical illness (23). Conversely, FGF-21 has been identified as having immunosuppressive effects including inhibition of CD8+ T-cell cytotoxicity (24), suggesting its effects may vary by context and concentration. This association should be treated as hypothesis-generating. Future research should test whether FGF-21 modulates vaccine-induced B- or T-cell memory formation.

I-FABP, a marker of small-intestinal enterocyte damage and component of EED, showed a negative but non-significant association with Ogawa seroconversion. The directional consistency of this trend supports the hypothesis that subclinical gut barrier disruption can dampen mucosal immune activation and contribute to variability in OCV performance, consistent with previous work linking EED to impaired oral vaccine responses (7, 8). Notably, I-FABP did not differ significantly between the pre-vaccination baseline and infected groups, suggesting that enterocyte damage may already be chronically elevated at baseline due to environmental exposure, with acute cholera not adding substantially further. Recent data from Lusaka have further documented associations between EED biomarkers and adverse outcomes in Zambian children (25).

This implies that gut integrity may be a chronic constraint on OCV immunogenicity in this setting, highlighting EED as a priority target for interventions aimed at improving oral vaccine performance.

Comparing the biomarker profiles of the pre-vaccination baseline group with those of individuals experiencing acute *V. cholerae* infection revealed substantial physiological divergence across multiple domains. Infected individuals had significantly lower ferritin and IGF-1, alongside significantly higher sTfR, CRP, AGP, and FGF-21. The concurrent fall in ferritin and rise in sTfR points to genuine iron depletion compounded by increased cellular iron demand, consistent with the anaemia of infection in which systemic inflammation drives macrophage iron sequestration (26). IGF-1 suppression reflects the well-documented inhibition of the growth hormone-IGF axis by systemic inflammation and catabolic stress (27). Thyroglobulin, HRP2, I-FABP, and sCD14 did not differ significantly between groups.

Correlation analysis demonstrated that acute infection substantially restructured inter-biomarker relationships. The pre-vaccination baseline showed sparse and modest associations, limited to AGP with CRP and AGP with sCD14. In contrast, the infected group exhibited a markedly expanded and stronger correlation network, converging inflammatory, micronutrient, iron metabolism, and gut integrity markers into a tightly co-regulated system. This contrast provides an important interpretive framework: it demonstrates that inter-biomarker relationships in the vaccinated cohort reflect relatively stable host characteristics, while the infection data illustrate the extreme co-perturbation that occurs during acute disease.

Several contextual factors warrant acknowledgement. Micronutrient deficiencies and protein-energy malnutrition, common in populations with limited dietary diversity, are associated with impaired immune function (9) and elevated baseline inflammation (28), potentially introducing residual confounding not fully captured by multivariable adjustment. EED, driven by chronic pathogen exposure in low-resource settings, may sustain elevated baseline inflammatory markers and impair absorptive capacity (10), Consistent with observations from other oral vaccine platforms, biomarker-based analyses suggest that chronic enteric dysfunction and altered nutritional-inflammatory states may contribute to heterogeneity in vaccine responses among individuals living in high-exposure environments (25). Co-infections with helminths, Helicobacter pylori, and malaria chronically elevate systemic inflammatory markers, potentially dampening vaccine-induced immunity (29). A Bangladeshi study demonstrated that children from lower socioeconomic backgrounds with elevated CRP and AGP had decreased vaccination effectiveness (30, 31). The Lukanga Swamps cohort, residing in a high-burden cholera-endemic environment, may carry a background of chronic immune activation that shapes pre-vaccination biomarker levels and contributes to inter-individual variability in seroconversion.

## 5 Strengths and limitations

Key strengths of this study include: (i) complementary quartile-based analyses allowing detection of non-linear biomarker–seroconversion relationships; (ii) simultaneous measurement of eleven biomarkers spanning gut integrity, systemic inflammation, micronutrient status, and metabolic function using the MEEDAT multiplex ELISA panel; (iii) inclusion of a comparator cohort of acutely infected individuals enabling direct comparison of pre-vaccination baseline profiles with those observed during active V. cholerae infection; and (iv) conduct entirely in a cholera-endemic population in Zambia, maximising ecological relevance.

Limitations include: (i) biomarkers were measured at a single pre-vaccination timepoint, not capturing dynamic fluctuations; (ii) the comparator cohort was relatively small, limiting statistical power; (iii) immune responses were assessed solely through vibriocidal antibody seroconversion, with mucosal endpoints such as intestinal IgA not measured.

## 6 Conclusion

Pre-vaccination host biomarker profiles, particularly FGF-21 and thyroglobulin, are associated with vibriocidal seroconversion after OCV revaccination in a cholera-endemic Zamian adult population.

Acute natural infection produces a qualitatively distinct multi-domain signature of nutrient depletion and acute-phase activation. These findings indicate that stable baseline host physiological status influences OCV immunogenicity and that the extreme perturbations of acute cholera represent an amplified form of the chronic host constraints that may limit vaccine responsiveness in endemic settings.

Collectively, our findings highlight host nutritional, metabolic, and inflammatory status at the time of vaccination as meaningful determinants of OCV immunogenicity. The extreme physiological disruption imposed by acute cholera likely represents an amplified form of the chronic subclinical perturbations and micronutrient insufficiency that constrain oral vaccine performance in endemic settings. Targeted nutritional or anti-inflammatory interventions prior to vaccination merit evaluation as strategies to improve OCV performance.

## Data Availability

The full datasets are not included in the article or **Supplementary Material**. Only aggregated summary statistics and results are presented. The underlying individual-level data are available from the corresponding author upon reasonable request, subject to institutional and ethical approvals. Requests to access these datasets should be directed to CC, Caroline.Chisenga@cidrz.org.
