# Supplementary material for: Pre-vaccination biomarkers of enteric dysfunction, inflammation and nutritional status associate with seroconversion to oral cholera vaccine and are perturbed during natural infection
Source: Front Immunol. 2026 Aug 14;17:1917668. doi: 10.3389/fimmu.2026.1917668 (PMC13522105; doi:10.3389/fimmu.2026.1917668)
Supplement: Supplementary file 1 [file Table1.docx]

**Supplementary Material**

**Baseline Biomarkers of Enteric Dysfunction, Inflammation, and Nutritional Status in Immune Responses to Oral Cholera Vaccine (OCV) and Natural Infection**

*This supplementary file contains Tables S1–S4 referenced in the main manuscript. Quartile boundary values are presented as the median of the relevant biomarker within each quartile.*

**Supplementary Table S1. Association of nutritional biomarkers with seroconversion by quartile.**

| **Biomarker** | **Inaba** | | | **Ogawa** | | |
| --- | --- | --- | --- | --- | --- | --- |
|  | **Total N=198** | **Seroconverted (n=100, 50.5%)** | **p-value** | **Total N=209** | **Seroconverted (n=110, 52.6%)** | **p-value** |
|  | n (% of total) | n (% of row total) |  | n (% of total) | n (% of row total) |  |
| **Ferritin, Median (IQR)** | 102.3 (47.8–330.6) | 102.3 (47.7–321.2) | 0.965 | 123.1 (49.9–330.6) | 140.2 (44.0–317.1) | 0.618 |
| Q1 (27.3 \| 26.6) | 54 (27.3) | 26 (48.1) | 0.969 | 53 (25.4%) | 31 (58.5%) | 0.445 |
| Q2 (78.4 \| 79.4) | 51 (25.8) | 27 (52.9) |  | 53 (25.4%) | 23 (43.4%) |  |
| Q3 (228.2 \| 228.2) | 38 (19.2) | 19 (50.0) |  | 42 (20.1%) | 23 (54.8%) |  |
| Q4 (530.1 \| 535.8) | 55 (27.8) | 28 (50.9) |  | 61 (29.2%) | 33 (54.1%) |  |
| **Tg, Median (IQR)** | 9.0 (5.5–16.3) | 8.5 (5.9–13.4) | 0.247 | 9.1 (5.6–16.4) | 7.8 (5.2–15.7) | **0.048*** |
| Q1 (4.0 \| 4.0) | 50 (25.3) | 22 (44.0) | **0.006*** | 52 (24.9%) | 31 (59.6%) | **0.003*** |
| Q2 (7.1 \| 7.0) | 50 (25.3) | 34 (68.0) |  | 52 (24.9%) | 35 (67.3%) |  |
| Q3 (11.9 \| 11.9) | 40 (20.2) | 23 (57.5) |  | 42 (20.1%) | 13 (31.0%) |  |
| Q4 (22.8 \| 23.2) | 58 (29.3) | 21 (36.2) |  | 63 (30.1%) | 31 (49.2%) |  |
| **sTfR, Median (IQR)** | 10.5 (7.1–14.1) | 10.4 (7.0–14.3) | 0.824 | 10.5 (7.2–13.9) | 10.0 (7.2–13.4) | 0.209 |
| Q1 (5.8 \| 5.8) | 54 (27.3) | 28 (51.9) | 0.931 | 54 (25.8%) | 29 (53.7%) | 0.520 |
| Q2 (9.1 \| 9.2) | 46 (23.2) | 23 (50.0) |  | 51 (24.4%) | 31 (60.8%) |  |
| Q3 (11.2 \| 11.2) | 37 (18.7) | 17 (45.9) |  | 39 (18.7%) | 19 (48.7%) |  |
| Q4 (16.5 \| 16.4) | 61 (30.8) | 32 (52.5) |  | 65 (31.1%) | 31 (47.7%) |  |
| **RBP4, Median (IQR)** | 2.5 (1.8–3.3) | 2.5 (1.7–3.3) | 0.967 | 2.5 (1.8–3.3) | 2.3 (1.8–3.3) | 0.295 |
| Q1 (1.4 \| 1.4) | 50 (25.3) | 27 (54.0) | 0.829 | 49 (23.4%) | 30 (61.2%) | 0.211 |
| Q2 (2.1 \| 2.1) | 51 (25.8) | 23 (45.1) |  | 54 (25.8%) | 30 (55.6%) |  |
| Q3 (2.8 \| 2.8) | 39 (19.7) | 20 (51.3) |  | 45 (21.5%) | 18 (40.0%) |  |
| Q4 (3.7 \| 3.8) | 58 (29.3) | 30 (51.7) |  | 61 (29.2%) | 32 (52.5%) |  |

*Tg, thyroglobulin; sTfR, soluble transferrin receptor; RBP4, retinol-binding protein 4. Quartile boundaries shown as median within quartile (Inaba | Ogawa). * p<0.05.*

**Supplementary Table S2. Association of growth-axis and EED biomarkers with seroconversion by quartile.**

| **Biomarker** | **Inaba** | | | **Ogawa** | | |
| --- | --- | --- | --- | --- | --- | --- |
|  | **Total N=198** | **Seroconverted (n=100, 50.5%)** | **p-value** | **Total N=209** | **Seroconverted (n=110, 52.6%)** | **p-value** |
|  | n (% of total) | n (% of row total) |  | n (% of total) | n (% of row total) |  |
| **Growth Factors** | | | | | | |
| **IGF-1, Median (IQR)** | 41.0 (13.6–97.8) | 36.8 (10.8–97.0) | 0.284 | 42.9 (15.1–100.3) | 34.4 (10.6–99.4) | 0.213 |
| Q1 (5.1 \| 5.1) | 50 (25.3) | 27 (54.0) | 0.765 | 50 (23.9%) | 33 (66.0%) | 0.064 |
| Q2 (25.5 \| 25.3) | 51 (25.8) | 27 (52.9) |  | 54 (25.8%) | 26 (48.1%) |  |
| Q3 (61.9 \| 61.8) | 37 (18.7) | 16 (43.2) |  | 39 (18.7%) | 15 (38.5%) |  |
| Q4 (135.7 \| 135.1) | 60 (30.3) | 30 (50.0) |  | 66 (31.6%) | 36 (54.5%) |  |
| **FGF-21, Median (IQR)** | 211.0 (100.2–449.6) | 198.8 (88.3–445.5) | 0.342 | 222.7 (100.6–460.3) | 200.4 (96.1–476.0) | 0.516 |
| Q1 (64.8 \| 64.6) | 51 (25.8) | 32 (62.7) | 0.202 | 52 (24.9%) | 31 (59.6%) | 0.317 |
| Q2 (144.0 \| 144.0) | 50 (25.3) | 22 (44.0) |  | 52 (24.9%) | 26 (50.0%) |  |
| Q3 (303.6 \| 303.2) | 41 (20.7) | 18 (43.9) |  | 43 (20.6%) | 18 (41.9%) |  |
| Q4 (705.6 \| 755.2) | 56 (28.3) | 28 (50.0) |  | 62 (29.7%) | 35 (56.5%) |  |
| **EED Markers** | | | | | | |
| **I-FABP, Median (IQR)** | 1,044.3 (669.4–1,682.0) | 979.1 (616.0–1,691.9) | 0.522 | 1,061.1 (678.8–1,716.8) | 979.1 (591.8–1,674.3) | 0.074 |
| Q1 (469.3 \| 484.4) | 52 (26.3) | 30 (57.7) | 0.678 | 53 (25.4%) | 32 (60.4%) | 0.370 |
| Q2 (852.1 \| 848.8) | 49 (24.7) | 23 (46.9) |  | 51 (24.4%) | 29 (56.9%) |  |
| Q3 (1,224.4 \| 1,256.2) | 38 (19.2) | 18 (47.4) |  | 41 (19.6%) | 19 (46.3%) |  |
| Q4 (2,233.2 \| 2,268.0) | 59 (29.8) | 29 (49.2) |  | 64 (30.6%) | 30 (46.9%) |  |
| **sCD14, Median (IQR)** | 1,768.6 (1,436.8–2,159.3) | 1,754.6 (1,378.1–2,250.1) | 0.840 | 1,759.6 (1,419.4–2,142.0) | 1,753.6 (1,375.1–2,127.4) | 0.573 |
| Q1 (1,183.6 \| 1,186.0) | 48 (24.2) | 28 (58.3) | 0.600 | 53 (25.4%) | 30 (56.6%) | 0.921 |
| Q2 (1,572.3 \| 1,582.4) | 50 (25.3) | 23 (46.0) |  | 52 (24.9%) | 27 (51.9%) |  |
| Q3 (1,885.8 \| 1,884.9) | 41 (20.7) | 19 (46.3) |  | 42 (20.1%) | 21 (50.0%) |  |
| Q4 (2,476.4 \| 2,482.6) | 59 (29.8) | 30 (50.8) |  | 62 (29.7%) | 32 (51.6%) |  |

*IGF-1, insulin-like growth factor 1; FGF-21, fibroblast growth factor 21; I-FABP, intestinal fatty acid-binding protein; sCD14, soluble CD14. Quartile boundaries shown as median within quartile (Inaba | Ogawa).*

**Supplementary Table S3. Association of systemic inflammation biomarkers with seroconversion by quartile.**

| **Biomarker** | **Inaba** | | | **Ogawa** | | |
| --- | --- | --- | --- | --- | --- | --- |
|  | **Total N=198** | **Seroconverted (n=100, 50.5%)** | **p-value** | **Total N=209** | **Seroconverted (n=110, 52.6%)** | **p-value** |
|  | n (% of total) | n (% of row total) |  | n (% of total) | n (% of row total) |  |
| **Systemic Inflammation** | | | | | | |
| **CRP, Median (IQR)** | 0.9 (0.3–3.1) | 0.6 (0.3–3.5) | 0.987 | 0.9 (0.3–3.0) | 0.8 (0.3–2.8) | 0.550 |
| Q1 (0.2 \| 0.2) | 52 (26.3) | 26 (50.0) | 0.575 | 52 (24.9%) | 29 (55.8%) | 0.859 |
| Q2 (0.5 \| 0.5) | 47 (23.7) | 27 (57.4) |  | 51 (24.4%) | 28 (54.9%) |  |
| Q3 (1.3 \| 1.3) | 38 (19.2) | 16 (42.1) |  | 44 (21.1%) | 23 (52.3%) |  |
| Q4 (6.0 \| 5.9) | 61 (30.8) | 31 (50.8) |  | 62 (29.7%) | 30 (48.4%) |  |
| **AGP, Median (IQR)** | 0.9 (0.6–1.2) | 0.9 (0.7–1.2) | 0.431 | 0.9 (0.7–1.2) | 0.9 (0.6–1.2) | 0.867 |
| Q1 (0.5 \| 0.5) | 52 (26.3) | 23 (44.2) | 0.635 | 51 (24.4%) | 29 (56.9%) | 0.534 |
| Q2 (0.8 \| 0.8) | 46 (23.2) | 24 (52.2) |  | 52 (24.9%) | 23 (44.2%) |  |
| Q3 (1.0 \| 1.0) | 38 (19.2) | 22 (57.9) |  | 42 (20.1%) | 24 (57.1%) |  |
| Q4 (1.6 \| 1.5) | 62 (31.3) | 31 (50.0) |  | 64 (30.6%) | 34 (53.1%) |  |

*CRP, C-reactive protein; AGP, α1-acid glycoprotein. Quartile boundaries shown as median within quartile (Inaba | Ogawa).*

**Supplementary Table S4. Baseline characteristics of the pre-vaccination baseline cohort and the acutely infected comparator cohort.**

| **Characteristic** | **Total N=263** | **Pre-vaccination Baseline (n=225, 85.6%)** | **Infected (n=38, 14.4%)** | **p-value** |
| --- | --- | --- | --- | --- |
|  | n (% of total) | n (% of row total) | n (% of row total) |  |
| **Sex** | | | | |
| Male | 115 (43.7) | 98 (85.2) | 17 (14.8) | 0.481 a |
| Female | 144 (54.8) | 127 (88.2) | 17 (11.8) |  |
| Missing | 4 (1.5) | 0 (0.0) | 4 (100.0) |  |
| **Age Category** | | | | |
| 15–25 | 69 (26.2) | 67 (97.0) | 2 (3.0) | 0.052 b |
| 26–45 | 85 (32.3) | 79 (93.0) | 6 (7.0) |  |
| 45+ | 71 (27.0) | 71 (100.0) | 0 (0.0) |  |
| Missing | 38 (14.4) | 8 (21.0) | 30 (79.0) |  |
| **HIV Status** | | | | |
| Negative | 218 (82.9) | 181 (83.0) | 37 (17.0) | **0.012 a*** |
| Positive | 44 (16.7) | 43 (97.7) | 1 (2.3) |  |
| Missing | 1 (0.4) | 1 (100.0) | 0 (0.0) |  |

*a Chi-squared test; b Fisher’s exact test. p-values calculated after excluding missing categories. * Statistically significant (p<0.05).*
